# The perceived impact of Covid-19 pandemic on the children with cerebral palsy: the parents’ perspective explored within the “6-F words” framework

**DOI:** 10.1186/s13034-023-00569-z

**Published:** 2023-02-15

**Authors:** Silvia Pizzighello, Marianna Uliana, Michela Martinuzzi, Matteo G. F. Vascello, Martina Cipriani, Martina Breda, Gianni De Polo, Andrea Martinuzzi

**Affiliations:** 1 Department of Conegliano, Scientific Institute IRCCS E. Medea, Via Costa Alta, 37, 31015 Conegliano, Treviso Italy; 2 King’s College London GKT School of Medical Education, London, UK; 3 Clinical Psychology Unit, ASST Papa Giovanni XXIII Hospital, Bergamo, Italy

**Keywords:** Covid-19, Cerebral palsy, ICF, Caregivers

## Abstract

**Background:**

In 2020 the world faced the spread of the coronavirus infection disease (Covid-19). This was a general public health emergency but many people with disabilities might have been particularly affected.

**Objective:**

This paper aims to investigate the impact of the Covid-19 pandemic on children with Cerebral Palsy (CP) and their families.

**Methods:**

110 parents of children with CP (aged 2 to 19) who completed a questionnaire were included. These children were under the care of one of the Italian Children Rehabilitation Centers. Socio-demographic and clinical information about patients and their families were collected. In addition, difficulties on adopting protective measures and in respecting lockdown rules by children were explored. We adopted the ICF (International Classification of Functioning, Disability and Health) framework to create multiple choice questions. Descriptive statistics were reported and logistic regression analyses were run in order to identify the predictors of perceived impairment in motor, speech, manual and behavioral abilities.

**Results:**

Daily activities of children, as well as rehabilitation and fitness sessions, underwent a change during the pandemic. Spending more time with family due to lockdown measures, has had, in some cases a positive effect however there was a perceived decrease in rehabilitation support and school activities. The age range (between 7 and 12 years) and difficulty in respecting rules emerged as significant predictors of the perceived impairment due to Covid-19 pandemic.

**Conclusions:**

The pandemic has had different impacts on children and their families on the basis of children’s characteristics. Rehabilitation activities during a hypothetic lockdown should consider these characteristics.

## Background

In March 12, 2020 the World Health Organization (WHO) declared the spread of the coronavirus infection disease (Covid-19) as a public health emergency and announced it as a pandemic. Covid-19 has been a worldwide health emergency requiring the adoption of several measures of containment, including a period of lockdown, social distancing and isolation to reduce the spread of the virus (WHO see) [1].

The adoption of lockdown measures, albeit necessary at the time, entailed adverse consequences in general health and access to health and social care services, household finances and the psychological well-being of children and their families [2]. The impact has been even more significant for children with physical or mental disabilities and their families, as shown by various studies which focused on this population [3–7].

Varengue [5] and Sutter [6] found that the physical wellbeing and mental health of children with disabilities was impaired due to the reduced availability of rehabilitative services and worsening of the caregivers’ mental health. For example, Dhiman [7] and Varengue [5] showed that the pandemic increased the stress, depression and the perception of stress on caregivers. The consequences varied depending on variables such as the children’ age and level of independence, whether a parent was the sole caregiver of the child, lack of confidence in telerehabilitation services and so on.

Among different disabilities of childhood, some studies focused on the impact of the pandemic on children and adolescents affected by cerebral palsy (CP) and on their families.

Akpinar [8] found that parents who did not help their children with physical exercises for different reasons, such as lack of self-confidence or older age, reported greater levels of anxiety. Anxiety was also associated with the age of the children, the motor and manual severity, the occurrence of comorbidities and parental income.

The inability of parents to perform home exercises during the reduction of rehabilitation services had a negative impact on the physical functioning of children, as demonstrated by Bashkar [9].

In general, authors found worsening of children's general health, mobility, spasticity, joint motion, social function, communication and mood [10, 11].

The level of activity, participation and environmental factors influenced the impact of the pandemic on body functioning in children with CP [12]. Similarly, Lai [13] found that Covid-19 reduced interactions, exercise participation and interesting in doing things. Moreover, it increased isolation and depression of adolescents and their families.

Many of these authors [14–16] highlighted the importance of telerehabilitation as a possible solution when regular and direct interventions by professionals are not possible.

During the Covid-19 pandemic Italy was one of the first countries to adopt a nationwide lockdown. On 9 March 2020 the government imposed restrictions on population movement unless for necessity, work and health circumstances. Additional measures included the temporary closure of non-essential shops and businesses.

To our knowledge there are no studies focused on the impact of the Italian lockdown on patients with CP. This paper aims to explore how the quarantine influenced the health of children with CP and their families. To this aim, we adopted the framework of the 6-F words (function, family, fitness, fun, friends, and future). The 6-F words were born in the World Health Organization’s International Classification of Functioning, Disability and Health (ICF) [17] context and they represent how, according to the ICF, we might think about health. The first word, ‘Function’ refers to what people do and considers the role, the job, the occupation of the participants and ‘play’ for children. It involves both ICF ‘Participation’ and ‘Activity’ categories. The second word ‘Family’ refers to the essential environment for children; in ICF terms, the family is the central ‘contextual factor’ in children’s lives. The third word ‘Fitness’ is the physical and mental well-being, and it involves both ‘Body structures’ and ‘Functions’ in the ICF framework. The words ‘Fun’ and ‘Friends’ include activities that people enjoy and relationships established with others; they both concern the ICF aspects of ‘Personal Factors’ and ‘Participation’. The word ‘Future’ considers the dreams and expectations of the parents for their children’s future and it covers all the aspects cited above.

By using a multiple choice questionnaire we aimed to explore the opinion of parents of children with CP on how lockdown measures affected the health of their children according to the ICF framework.

## Methods

### Protocol

This cohort study was addressed to all the parents of children with cerebral palsy who attended one of the Italian Children Rehabilitation Network centers in the North-Eastern Italian Region of Veneto between July 2021 and January 2022. This network provides pediatric specialty care and patients are discharged once they reach 18 years of age. Inclusion criteria for the study were: (1) diagnosis of CP; (2) ability to speak/read/understand Italian; (3) to be under the care of one of the Children Rehabilitation Network centers between July 2021 and January 2022.

The study has been reviewed and approved by our center Ethics Committee (Prot. 994-CE). It is adherent to the committee’s recommendations and was conducted in accordance with the ethical standards of the Declaration of Helsinki (1964).

The questionnaire was designed so that it could be self-administered or administered by proxy depending on the parents’ decision. Parents completed a questionnaire for each child. The first part of the questionnaire was intended to collect psychosocial information about the participant (sex, age, education, extension of the motor impairment) and their parents (marital and employment status before and during Covid-19). In addition to this, any difficulty of adopting protective measures (as masks, glove, etc.) and respecting lockdown rules by children were explored.

The second part of the questionnaire explored the occurrence of any possible changes in the motor, speech, manual and behavioral abilities of children during lockdown by using a Likert scale that provided seven possible answers: -3 ‘severely impaired’, -2 ‘quite impaired’, -1 ‘little impaired’, 0 ‘none’, + 1 ‘little improved’, + 2 ‘quite improved’ and + 3 ‘very improved’.

Finally, the occurrence of any type of change in the six areas of the ‘6-F words’ [18] was explored by using multiple-choice questions.

### Data analysis

Descriptive statistics, i.e. frequency and percentages for categorical variables, median and interquartile range for continuous variables were employed to describe variable distributions.

We then ran seven logistic regression analyses in which the perception of any possible impairment in the motor, speech, manual and behavioral abilities of the participants were entered as the target variable and children’s and parents’ characteristics, difficulty in adopting protective measures and in respecting rules were entered as predictive variables. In order to do this, we computed a dichotomous variable (Yes/No) in which the level “Yes” included the scores from -3 to -1 and the Level “No” included the scores from 0 to + 3.

### The Covid impairment score

In order to obtain more information concerning the severity of the perceived impairment in the motor, speech, manual and behavioral abilities of children, we then created a global index (*Covid impairment Score*) by summarizing the absolute scores for each of the four abilities when the scores were − 3, − 2 or − 1. Scores from 0 to 3 were not considered since they do not represent the perception of an impairment. The *Covid impairment score* represented the severity of the impairment and it ranged from 1 (absolute score “1” in only one ability; little impairment in one ability) to 12 (absolute score “3” in all four abilities; severe impairment in all four abilities).

## Results

### Patients

The parents of 110 children (62 M, 48 F) aged 2 to 19 (Median 10.00, IQR 6.25–15) completed the questionnaire and were included in the study. Informed consent was given by all participants. Characteristics of the participants are reported in Table 1.

**Table 1:** Children’ characteristics: age range, sex, extension of the motor impairment and years of education

Age range	
0–6 years	28 (25.45%)
7–12 years	42 (38.20%)
13–19 years	40 (36.36%)
Sex	
M	62 (56.36%)
F	48 (43.64%)
Extension of the motor impairment	
Diplegia	26 (23.64%)
Hemiplegia	45 (40.91%)
Quadriplegia	37 (33.64%)
Not defined	2 (1.82%)
Education	
0–5 years	57 (51.82%)
6–8 years	13 (11.82%)
more than 8 years	25 (22.73%)
Not defined	15 (13.64%)

Information concerning the parents’ characteristics (marital status, employment status before and after Covid-19) were collected and are reported in Table 2.

**Table 2:** Parents’ characteristics: marital status, employment status before and during the Covid-19 pandemic

Marital status of parents	
Married	85 (77.27%)
Separated	4 (3.64%)
Divorced	2 (1.82%)
Cohabitants	16 (14.54%)
Widowed	1 (0.91%)
Not disclosed	2 (1.82%)
Employment status pre Covid-19	
Both employed	63 (57.27%)
Both unemployed	3 (2.73%)
One employed	40 (36.36%)
Retirement	0
Not disclosed	4 (3.64%)
Modification in the employment status of parents during Covid-19 (more than one answer)	
None	83 (75.45%)
Unemployment benefit	7 (6.36%)
Benefit for freelance jobs	8 (7.27%)
Work from home	18 (16.36%)
Work schedule changes	13 (11.82%)
Loss of job	5 (4.55%)

Parents were then asked to assess if their children had any difficulties adopting protective measures (for example wearing gloves or face masks). Forty-nine percent reported no difficulties, 16.36% reported difficulties and 33.6% claimed that the use of protective measures was not mandatory for their children and therefore they did not apply them.

Concerning the respect of the rules by the children (for example keeping social distancing or respecting lockdown), about 71% reported no difficulties whereas about 27% found it difficult and about 4% gave no answer. The difficulty in respecting rules was independent from the age group (χ^2^(2) = 3.54, p = 0.17).

### Regression analyses

We ran seven logistic regression analyses in which the perception of impairment due to Covid-19 (Yes/No) was entered as the target variable and the following variables as predictors: the child’s sex, age, and diagnosis, parent’s marital and employment status, difficulty in adopting protective measures and difficulty in respecting rules. Results are presented in Table 3.

**Table 3:** Results of logistic regression analysis

Variables	Odds ratio	Estimate (95% CI)	p value
Sex	0.68	0.32–1.46	0.32
Age			
0–6 yrs	Ref		
7–12	3.80	1.38–10.50	< 0.01^a^
13–19	1.73	0.63–4.74	0.29
Diagnosis			
Diplegia	Ref		
Hemiplegia	1.01	0.37- 2.76	0.99
Quadriplegia	0.69	0.26–1.81	0.45
Parents’ marital status			
Married or Cohabitants	Ref		
Separated or divorced	0.76	0.16–3.59	0.73
Employment status			
Both employed	Ref		
Both unemployed	0.71	0.06–8.26	0.79
Only one parent employed	2.13	0.95–4.79	0.07
Difficulty in adopting protective measures			
None	Ref		
Yes	1.57	0.53–4.66	0.41
Not applicable	0.76	0.33–1.77	0.53
Difficulty in respecting rules			
None	Ref		
Yes	12.30	3.88–38.80	< 0.01^a^

^a^significant result

Two significant predictors of the perception of impairment due to Covid-19 emerged: age between 7 and 12 years and difficulty in respecting rules. The Akaike information criterion (AIC) of the final model with these two variables was 126. The receiver operating characteristic (ROC) curve shows that the predictive accuracy within the logistic regression model was satisfactory (AUC = 0.79).

### Covid impairment score

**Fig. 1:**
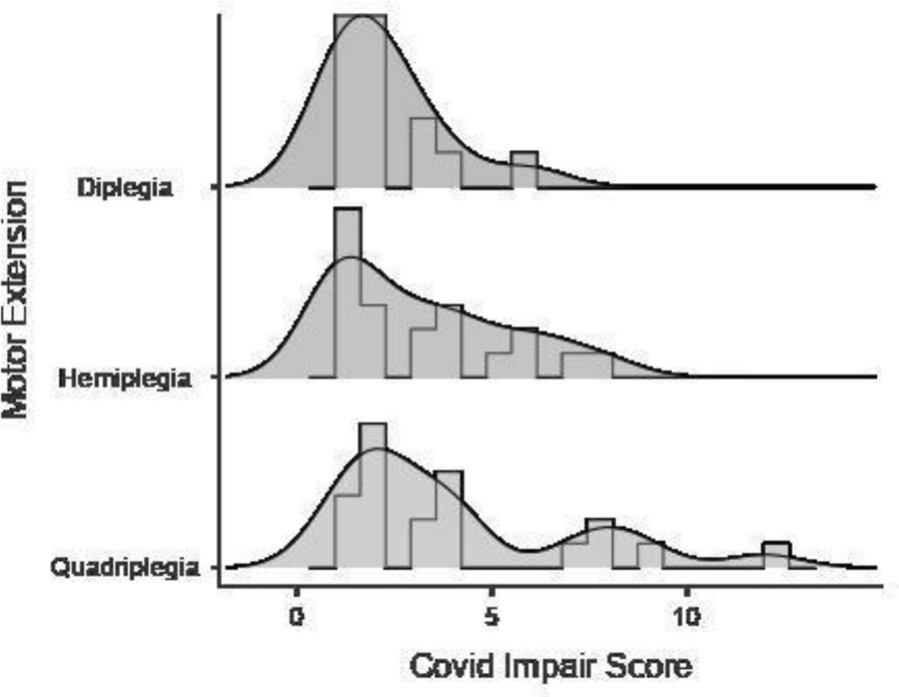
Distribution of *Covid impairment score* depending on the extension of motor impairment

The median of the *Covid impairment scor*e was 2, the interquartile range was 1–4. Only one quarter of the sample reached a score greater than 4. Depending on the extension of the motor impairment, a different distribution of the *Covid impairment scor*e was observed (see Fig. 1).

With respect to the different abilities, 46 parents out of 110 claimed an impairment on the children’s behavior, 36 claimed an impairment in motor functioning, 18 and 11 observed an impairment in the manual and the speech functioning respectively. Levels of impairment of each ability are reported in Table 4.

**Table 4:** Different levels of impairment of each ability

	Motor	Speech	Manual	Behavior
Little impaired	23 (63.89%)	5 (45.45%)	9 (50%)	28 (60.87%)
Quite impaired	7 (19.44%)	4 (36.36%)	6 (33.33%)	11 (23.91%)
Severely impaired	6 (16.67%)	2 (18.18%)	3 (16.67%)	7 (15.22%)
Total	36	11	18	46

The table reports frequencies and rates (in bracket)

### The 6-F words

The impact due to the pandemic on the 6-F words (function, family, fitness, fun, friends, and future) was explored. Results for each area are presented below.

### Function

Activities of children underwent a change both at home and at school due to the pandemic and its associated restrictions. Half of the participants (45.5%) reported changes in daily activities; of these, 34% specified that this was towards an impairment. Behaviors concerning eating and drinking changed in 14.5% of cases, with mainly an increase in food intake and in the consumption of junk food. Habits concerning personal hygiene changed in 12% of cases but this resulted mainly in an improvement. Dressing skills and toileting changed in less than 10% of cases.

For more than half of the children (61%) the frequency of school activities was reduced and completely interrupted for about one-third of them (27.3%). Forty-two percent reported a reduced availability of the support teacher and a complete interruption of support in about 26% of cases. Opportunities for leaving the house decreased in 23.6% of participants.

### Fitness

For about one-third of children (27%) a temporary interruption (often from 1 to 4 months) of rehabilitative activities was necessary due to restrictions. For those who had access to rehabilitation sessions, their weekly frequency was reduced in 16.3% of cases, while the duration of their sessions was decreased for less than 3% of participants. Only eight participants continued their rehabilitative sessions in different formats or modalities, for example via telehealth. Furthermore, medical assessments and treatments were temporarily interrupted in 21% of cases. Only urgent treatments and assessment where guaranteed (19% of cases).

The physical activities done during leisure time were interrupted in 34.55% of cases. A decrease in the frequency of sessions occurred in 31% of cases whereas the duration of each session was reduced in less than 10% of cases. During the pandemic about 10% of parents found alternative ways to maintain their children’s physical activities: mainly by outdoor activities or by using assistive devices or sporting equipment.

### Family

Most families (about 74.5%) increased the time they spent together as a consequence of restrictions and decreased opportunities to leave the house. During this time, the family's mood was perceived as changed in 66.3% of cases: out of this percentage, half of participants perceived an improvement; one third perceived a worsening and about 20% perceived both a worsening and an improvement. The worsening involved all members of the family for 13.6% of cases and between siblings for 12% of the cases; relationships between the child and parents or between the couple were less involved (9% and 8.2%, respectively). When the family mood improved, this equally occurred between child and parents and between siblings (25.45% and 20%, respectively).

Despite the temporary complete interruption of school and rehabilitative activities, only 14.5% of families received home support or support from specialist staff, and when this occurred the service was mainly in the form of private support paid for by the family itself.

### Fun and friends

The amount of free time did not change for half of the children participating in the study (43.6%), it was reduced for about 17.3% of participants and it increased in 15.45% of cases. About one-third of families reported their children choosing new activities to entertain themselves (such as trying new hobbies, watching new series, or YouTube channels…). Due to the necessity of adopting protective measures, the opportunities to meet friends were reduced for most of the children (62%) but 14.5% of them adopted alternative measures of meeting (as video calls). The frequency of meetings did not change in 33.6% of cases whereas it increased in less than 2% of cases.

### Future

Expectations concerning the future of the children's rehabilitation and fitness, the family’s mood, leisure time, and social relationships were explored via open questions. A qualitative analysis showed the following results. About 65% of children’s parents hoped that rehabilitation activities returned as to how they were before the pandemic and they wished that their children’s fitness would return to previous levels or even improve (38.18% and 40%, respectively). Around 44% of parents wished to improve their family’s mood, however, 33.3% expressed satisfaction with the post-Covid-19 situation and wished to maintain it. Most of the parents hoped to improve the quality of their children’s leisure time (26%) or at least to recover it. Similarly, 48% and 52%, would like to increase the number of their children’s relationships or at least to recover them.

## Discussion

In this paper, parents of more than one hundred children with CP gave their opinion in regard to the global impact of the Covid-19 pandemic on the health of their children as described in the ICF. The group of children was mainly composed of patients aged from 7 to 19 years and the most prevalent diagnosis was hemiplegia, followed by quadriplegia and diplegia. Most of the parents were married or cohabiting (less than 7% were separated, divorced or widowed). In about 95% of cases, one or both parents were employed before the pandemic but about 5% of them lost their job due to the pandemic. More frequently, parents were required to work from home or to accept work scheduling changes whereas less than 15% received job benefits.

During the pandemic, the Italian government made mandatory the use of certain measures in order to protect people from the spread of the virus. The adoption of these precautions may have been difficult to follow for certain groups of patients; among the participants 16% of parents reported their children had difficulties in adopting protective measures whereas about 50% of the group encountered no difficulty. About one third of children were excluded from these mandatory precautions due to the incompatibility of their disability with the use of precautions such as masks or gloves. More often, parents observed that their children had difficulties in respecting rules, independently from the age range.

The age range together with the difficulty in respecting rules were the two significant predictors of the perception of impairment due to Covid-19 pandemic.

For about six children out of ten aged between 7 and 12, parents reported a general impairment due to the pandemic; this rate reduced for children aged 13- 19 (four out of ten) and further reduced when considering the youngest, aged between 0 and 6 (three out of ten). The age of children with CP has been studied only in relation to the parents’ mood during the pandemic but with opposite results: Akpinar [8] found a positive relationship between the child’s age and the State Anxiety (STAI-1 Scale) whereas Dhiman [7] found that parents of children younger than 6 years experienced more depression and anxiety when compared to parents of children older than 6 years.

About 9 parents out of ten who observed their children having difficulties in respecting rules, then claimed a negative impact on the child’s physical and mental health due to the pandemic.

Concerning the children’s abilities (motor, speech, manual and behavior) the *Covid impairment score* reached higher levels in children with more extended motor impairment (quadriplegia), thus suggesting that these patients could be more affected by the interruption of health services. This is in accordance with Biyik [12] who found that children scoring Gross Motor Functioning Classification Scale (GMFCS) [19] levels IV and V (children with limited self mobility or transported in a manual wheelchair) presented increased tonus and a decreased range of motion. Moreover, Akpinar [8] observed that parents of children with higher levels of GMFCS claimed greater levels of anxiety. Considering the children’s abilities, behavior and the motor functioning were more affected during the pandemic. In accordance with this, different authors found a decrease in the general mood [13] or an increase in irritability, aggressiveness and anger [9] and, more generally, behavioral problems [5, 10, 12]. This was true also for children with other disabilities [20]. Similarly, motor functioning was reported as one of the more affected areas in children with CP [9, 10, 12] as well as other disabilities [6].

The impact of the pandemic on the health of children as intended in the ICF was explored. One child out of two was forced to change their daily routine both at home and at school. One child out of two also suffered some reduction in rehabilitation, even if temporary. Only one parent out of ten reported being able to find alternative ways to maintain the physical activities of their children. More than half of parents wished that their children’s fitness level and their rehabilitative activities returned to levels prior to Covid-19. In most cases the time that families spent together increased and this represented a positive aspect for several families, as demonstrated by the fact that about one third of parents were satisfied with this situation and would like to maintain it. Faccioli et al. [21] in their study exploring a population with disabilities found that “the increased time spent with family members was judged positively by 27.2% of parents and by 64.2% of adolescents”.

In opposition to these results, the opportunities of children to meet friends was reduced for many children, so much that about one child out of two would like to increase the number of relationships or at least to recover them after the pandemic, as reported by parents.

## Conclusions

One parent out of two observed some impairment due to COVID-19 in the health of their children with CP, but most parents claimed low levels of impairment (low Covid impairment scores). This occurred mainly on motor and behavioral abilities. This study also suggests that the pandemic has had a different impact on children and their families on the basis of certain characteristics such as age and type of motor impairment. Therefore, in the case of another pandemic, there can be increased awareness regarding the child's diagnosis and severity of impairment. Parents of children with CP and other disabilities should be made aware of other available resources that can be used to blunt the impact of the pandemic on their health. Increased awareness of telehealth, pamphlets that help parents perform exercises with their children, virtual support groups are all suggestions that can lighten the burden. Moreover, it would be useful to differentiate the rehabilitative proposal on the basis of child’s characteristics and to give some support to promote the acceptance of new rules and measures required by the pandemic.

This study has the following limitations: we did not consider the potential spread of Covid-19 in the families; we did not use specific scales in order to measure the levels of abilities (as for example, GMFCS); we initially had a drop out of 80% of participants who, for several reasons, did not agree with the participation. Furthermore, the sample might not be representative of the general population of families with children with CP as it only included children under the care of a tertiary rehabilitations service. Finally, the study was conducted 1 year after the first wave of Covid-19 in Italy and our results can therefore represent the long-term perception of Covid-19 pandemic on participants.

## Data Availability

The datasets obtained and analyzed during the current study are available from the corresponding author on reasonable request.

## Abbreviations

CP: Cerebral palsy
ICF: International Classification of Functioning, Disability and Health
WHO: World Health Organization
AIC: Akaike information criterion
ROC: Receiver operating characteristic
AUC: Area under the curve
STAI-1: State anxiety scale
GMFCS: Gross motor functioning classification scale
